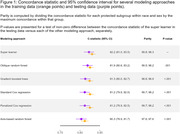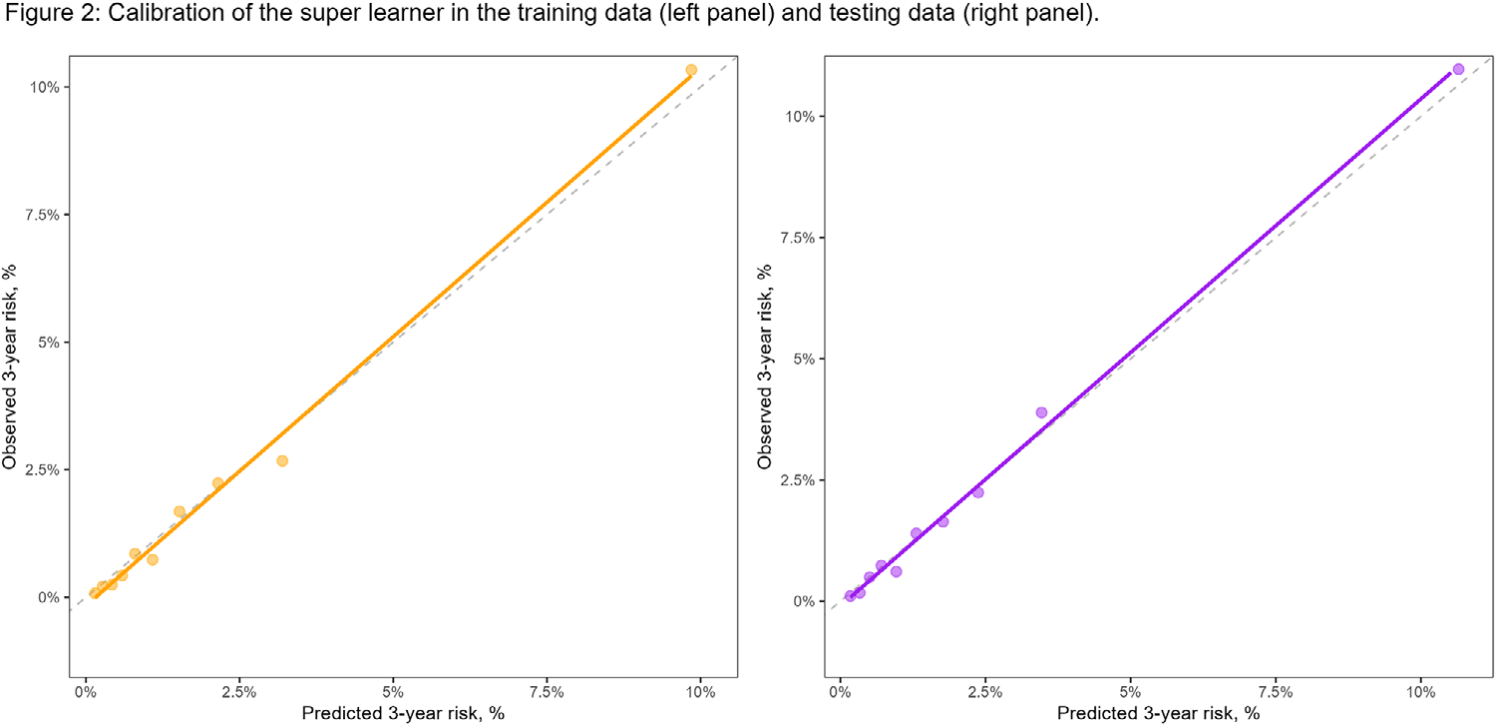# Screening for the Spectrum of Cognitive Impairment in Primary Care: Development of a Machine Learning Informed, Workflow‐Friendly Care Model

**DOI:** 10.1002/alz.095728

**Published:** 2025-01-09

**Authors:** Jeff D. Williamson, Byron C Jaeger, James R. Bateman, Marc D. Benayoun, Amy Boehm, Maryjo Cleveland, Eric D Kirkendall, Michelle M. Mielke, Nicholas M. Pajewski, Joseph D Rigdon, Heather M Snyder, Matthew G. Weiss, Paul D Yelton, Suzanne Craft

**Affiliations:** ^1^ Wake Forest University School of Medicine, Winston‐Salem, NC USA; ^2^ Wake Forest University School of Medicine, Winston Salem, NC USA; ^3^ Alzheimer’s Association, Chicago, IL USA; ^4^ Wake Forest University School of Medicine, Advance, NC USA

## Abstract

**Introduction:**

Over 9 million Americans are projected to have dementia by 2030, and adults with mild cognitive impairment (MCI), a potential pre‐cursor to dementia, will also rise. With recent and emerging clinical trial evidence for interventions to slow the progression of MCI to dementia, identification of persons in primary care with undiagnosed early‐stage cognitive impairment may provide opportunity for preventive intervention.

**Methods:**

A Machine Learning (ML)‐based prediction model trained using data from the electronic health record was applied to patients without formal diagnoses of cognitive impairment who were currently seen in selected primary care practices. The ML model is a super learner ensemble of prediction models, combining predictions from boosted trees, oblique random forests, and Cox regression trained using patient records from 2017‐01‐01 to 2018‐12‐31. We externally validated the super learner’s discrimination, calibration, and fairness using records from 2019‐01‐01 to 2020‐12‐31 (no overlapping patients). Patients for whom the model triggered an alert were recommended by their primary care practitioner to undergo cognitive assessment, consisting of a 30‐minute remotely‐administered tablet‐based cognitive battery. Patients also underwent blood collection for AD biomarkers (ptau217, Ab42, neurofilament light, GFAP), and neuroimaging (MRI, amyloid PET).

**Results:**

In the testing cohort, the super learner obtained a concordance statistic (95% confidence interval) of 0.822 (0.810, 0.835), higher than any of the individual ML models (Figure 1), and its predicted risk aligned with observed risk in calibration slope plots (Figure 2). Results of cognitive testing plus expert adjudication of the first 75 patients will be presented by proportion of patients adjudicated as not impaired versus MCI. We will also report categories of therapy such as behavioral (sleep, exercise, etc.), medical (hypertension control, alcohol cessation), or pharmacologic (anti‐amyloid therapy) that were recommended based on cognitive, imaging, biomarker, and medical findings. Also reported will be initial feedback on workflow and provider satisfaction with this screening and care model from primary care providers.

**Conclusion:**

While predictive models of undiagnosed cognitive impairment are imperfect, they can facilitate scalable and feasible primary care screening models leading to effective care plan design for patients with early cognitive impairment and without adversely affecting workflow.